# Primary Health Care and the Third Sector in the face of violence between intimate adolescent partners*

**DOI:** 10.1590/1518-8345.3811.3341

**Published:** 2020-09-07

**Authors:** Rafaela Gessner Lourenço, Rosa Maria Godoy Serpa da Fonseca

**Affiliations:** 1 Universidade Federal do Paraná, Departamento de Enfermagem, Curitiba, PR, Brazil.; 2 Scholarship holder at the Coordenação de Aperfeiçoamento de Pessoal de Nível Superior (CAPES), Brazil.; 3 Universidade de São Paulo, Escola de Enfermagem, São Paulo, SP, Brazil.

**Keywords:** Intimate Partner Violence, Adolescent, Gender-Based Violence, Primary Health Care, Primary Prevention, Qualitative Research, Violência por Parceiros Íntimos, Adolescente, Violência de Gênero, Atenção Primária à Saúde, Prevenção Primária, Pesquisa Qualitativa, Violencia de Pareja, Adolescente, Violencia de Género, Atención Primaria de Salud, Prevención Primaria, Investigación Cualitativa

## Abstract

**Objective:**

to identify and analyze the perceptions and practices of health professionals and of the third sector regarding adolescence and violence between intimate adolescent partners.

**Method:**

an exploratory and descriptive study. Data was collected in two sessions of a Critical-Emancipatory Work Workshop carried out with 55 professionals who work in Primary Health Care services and services related to the third sector. Data was submitted to content analysis with the support of the WebQDA software. The categories of analysis used were gender and generation.

**Results:**

the perceptions and practices in the face of violence between intimate adolescent partners are based on common sense and subjugate female adolescents in particular. In the health services, violence is perceived during consultations for other demands, under the biomedical paradigm. Third sector professionals understand the confrontation of violence as a responsibility of the health area.

**Conclusion:**

negative and stereotyped conceptions of violence between intimate adolescent partners are marked by gender and generation biases. The study highlights the need to promote networking actions in the professional practices that consider the historical and social understanding of adolescence to cope with the problem.

## Objetivo

identificar e analisar as percepções e as práticas de profissionais da saúde e do terceiro setor a respeito da adolescência e da violência entre parceiros íntimos na adolescência.

**Método:**

estudo exploratório e descritivo. Os dados foram coletados em duas sessões de uma Oficina de Trabalho Crítico-emancipatória desenvolvida com 55 profissionais que atuavam em serviços da Atenção Primária à Saúde e serviços vinculados ao terceiro setor. Os dados foram submetidos à análise de conteúdo com apoio do software WebQDA. As categorias de análise utilizadas foram gênero e geração.

**Resultados:**

as percepções e as práticas diante da violência entre parceiros íntimos adolescentes são balizadas pelo senso comum e subjugam, sobretudo, as adolescentes do sexo feminino. Nos serviços de saúde, a violência é percebida no momento de atendimento a outras demandas, sob o paradigma biomédico. Os profissionais do terceiro setor entendem o enfrentamento da violência como responsabilidade da área da saúde.

**Conclusão:**

as concepções negativas e estereotipadas sobre a violência entre parceiros íntimos adolescentes são marcadas por vieses de gênero e geração. O estudo evidencia a necessidade de promover ações em rede nas práticas profissionais que considerem a compreensão histórica e social da adolescência para o enfrentamento do problema.

## Abstract (es)

**Objetivo:**

identificar y analizar las percepciones y prácticas de los profesionales de la salud y del tercer sector con respecto a la adolescencia y a la violencia entre parejas en la adolescencia.

**Método:**

estudio exploratorio y descriptivo. Los datos se recopilaron en dos sesiones de un Taller de Trabajo Crítico-Emancipador desarrollado con 55 profesionales que actuaban en servicios de Atención Primaria de Salud y servicios vinculados al tercer sector. Los datos se sometieron a análisis de contenido con la utilización del software WebQDA. Las categorías de análisis utilizadas fueron género y generación.

**Resultados:**

las percepciones y prácticas sobre la violencia entre parejas adolescentes se guían por el sentido común y abruman, sobre todo, a los adolescentes del sexo femenino. En los servicios de salud, la violencia se percibe cuando se atienden otras demandas, bajo el paradigma biomédico. Los profesionales del tercer sector entienden el afrontamiento de la violencia como una responsabilidad sanitaria.

**Conclusión:**

las concepciones negativas y estereotipadas sobre la violencia entre parejas adolescentes están marcadas por sesgos de género y generación. El estudio destaca la necesidad de promover acciones en red en las prácticas profesionales que consideren la comprensión histórica y social de la adolescencia para afrontar el problema.

## Introduction

Violence, in its structural, communitarian, extra or intra-family involvement, affects the adolescent population in practically all geopolitical spaces. An important form of violence, which generates destructive consequences for the lives of adolescents in situations of violence and perpetrators, is that which occurs in affective or sexual relationships, called Violence between Intimate Partners (*Violência entre Parceiros Íntimos*, VPI) in adolescence^(^ 1 ^)^.

A study conducted with 3,200 Brazilian adolescents revealed that 86.9% of those interviewed had suffered some form of violence during an affective or sexual relationship. The forms of violence most observed were verbal violence (85.0%), followed by sexual violence (43.8%), threats (24.2%), and physical violence (19.6%). In addition, more than 90% of these adolescents consider the different manifestations of the VPI as a serious phenomenon^(^ 2 ^)^. On the international scene, other studies^(^ 3 ^-^ 4 ^)^ corroborate the importance of the VPI approach in adolescence in different political, economic, and social contexts.

VPI in adolescence is determined by generational, historical, economic issues and, above all, by the experience of gender roles that polarize different tensions for the genders, such as the production and reproduction of socially accepted models^(^ 5 ^)^.

Despite the relevance of this issue, the health practices directed at adolescents in Primary Health Care (PHC) services do not usually incorporate its confrontation. VPI situations are often let out of the health field range and are treated as situations restricted to the personal lives of those involved. Other difficulties in the sector are the structural insufficiency of the health equipment for the care of adolescents in situations of violence and the difficulty of acting through a network. Also, further difficulties are driven by generational issues that adolescents encounter in accessing the health services^(^ 6 ^-^ 7 ^)^.

In Brazil, with the expansion of the neoliberal trend that has also struck the Public Policies aimed at the adolescent population, since 1980, the Third Sector’s participation grew up in the implementation of projects executed mostly by Non-Governmental Organizations (NGOs) that congregate these characteristics: they are private, non-profit entities, able to manage their activities on a voluntary basis^(^ 8 ^)^. 

In this context, research shows the potential of third sector institutions, in partnership with PHC, to act as spaces for promoting health and social inclusion, with a view to providing adolescents with autonomy and improving their health-disease process^(^ 9 ^-^ 10 ^)^; therefore, being characterized as an alteration to the network confrontation of the adolescent VPI. Despite this possibility, there is a knowledge gap in research identifying actions aimed at adolescent VPI established between PHC and Third Sector services^(^ 6 ^)^.

In order to understand the possibilities of confronting adolescent VPI, it is necessary to know how this problem is perceived and faced by the professionals from the various institutions in the territories where they operate. With this in view, we wonder: What is the perception of health and third sector professionals about violence in affective and sexual relationships among adolescents? What are the practices of these professionals regarding adolescent VPI? To answer these questions, this study had as objectives to identify and analyze the perceptions and practices of health and third sector professionals regarding adolescence and violence between intimate partners in adolescence, in the light of gender and generation categories.

The relevance of this study lies in the possibility of increasing scientific knowledge on adolescent VPI for the health field and to contribute to the formulation of public policies for the training and qualification of health professionals and other areas, through the articulation of confronting the phenomenon to the categories of gender and generation analysis.

This study considers that the gender category is inherent to social relationships, and it is also primordial for the construction of meanings about the power relations between men and women, men and men, and women and women^(^ 11 ^)^. In turn, the generation category establishes the space occupied by the different generations, which suffer the impact of society’s events. More than an age concept, the generation marks the groups’ social statutes by the political and ideological similarities that differentiate them from others, in time and space^(^ 12 ^)^. These categories, as a social construct, determine the social place and the experience of affective and sexual relationships between adolescents.

## Method

An exploratory and descriptive study with a qualitative approach, whose theoretical framework is based on the Theory of Praxis Intervention of Nursing in Collective Health (*Teoria da Intervenção Práxica da Enfermagem em Saúde Coletiva*, TIPESC). The theory turns to research and nursing intervention in the collective health-disease process and presents the following stages: 1) capturing objective reality; 2) interpreting objective reality; 3) constructing the project of intervention in the objective reality; 4) intervening in the objective reality; and 5) reinterpreting objective reality. This study met the two first stages of the TIPESC^(^ 13 ^)^, dedicated, mostly, to the recognition and understanding of the social phenomena. 

The study was carried out in the municipalities of Curitiba and São Paulo. In the municipality of Curitiba, the scenarios were in PHC services with and without Family Health Strategy (FHS) of two administrative regions, besides a service linked to the third sector in one of these regions. In São Paulo, the research scenarios were a health service linked to the FHS’s PHC and a service linked to the third sector in the same territory. In both cities, the third sector’s services surveyed were NGOs that developed activities of social assistance, education, sports, theater, music, digital inclusion, and professional training courses with adolescents from 12 to 18 years old. 

Data was collected between November 2016 and July 2017 from two sessions of a Critical-emancipatory Work Workshop (*Oficina de Trabalho Crítico-emancipatória*, OTC), lasting three hours each, developed in each of the surveyed scenarios, totaling ten sessions. OTC is based on critical-emancipatory education and on the dialectic method of exposure and analysis. It encompasses the moments of warm-up, individual reflection, group reflection, and synthesis as triggers of each process of collective construction^(^ 14 ^)^. Each session took place based on a script developed and tested by the researchers to understand the perceptions and practices of health professionals and of the third sector on violence between intimate adolescent partners. They occurred in the investigated services, in private rooms, and during the working hours of the participants. 

The participants for the OTCs were indicated by key informants^(^ 15 ^)^ of the territories (health service managers and coordinators of third sector institutions) for their relevance in serving adolescents in the investigated service. There was no refusal to participate in the study from the invited individuals.

The following were defined as inclusion criteria: being a health professional (as set forth in the National Basic Care Policy) and acting, even if indirectly, in the health care of adolescents in the same scenarios, or being a third sector professional and acting, even if indirectly, in the care of adolescents in the same scenarios.

The data produced in the workshops were recorded, transcribed in full, and analyzed according to Bardin’s proposition^(^ 16 ^)^ to identify the empirical categories, which were discussed in the light of the gender and generation analysis categories. In this study it was decided to transcribe all the moments of the OTC sessions developed, making a total of 32 hours of recording. 

The WebQDA software was used to support qualitative data analysis due to its relevance to the organization and analysis of non-numeric and unstructured data^(^ 17 ^)^. The participants’ speeches were identified by their name’s first letters, followed by the service and scenario in which they served.

Besides the speeches, images composing a collage panel were analyzed, which was built by the participants as a result of an OTC activity to understand the participants’ perception of the adolescent. These images were public, as they were taken from two national journals.

The study complied with the legal provisions of Resolution No. 466/2012 from the National Health Council Resolution and was approved by the Research Ethics Committee of the University of São Paulo’s Nursing School and the Ethics Committees of the Municipal Health Secretariats of Curitiba and São Paulo.

Following methodological rigor, the criteria established for the Reporting Qualitative Research (COREQ) were observed in the elaboration and development of the research.

## Results

In the Curitiba scenario, 34 professionals participated; of these, 25 were from the health team (eight nurses, three dentists, one nurse assistant, two nursing technicians, one oral health assistant, three physical educators, two physiotherapists, three physicians, and two psychologists) and nine from the third sector (one social worker, three social projectors, one pedagogical coordinator and three teachers). In São Paulo, 21 professionals participated, 10 of whom were from the health team (three nurses, one dentist, one nurse technician, one physician, two Community Health Agents (CHAs), one social worker and one pharmacy technician) and 11 from the third sector (one social worker, two pedagogical coordinators, four teachers, and four management assistants).

The data from the group speeches and panels produced in the OTCs guided the coding process through the WebQDA software. Ten data sources were created out of the transcript files from each of the OTC sessions carried out and the images of the produced panels, using the internal sources import tool. In all, 182 images were inserted in the panels and 131 were analyzed since the 51 excluded images did not concern the concepts indicated in the proposed activity. 

After exhaustive reading of the transcripts and analysis of the images, the information that highlighted the core meaning of the communications were coded in 52 free codes, i.e., codes that bind ideas without a hierarchical system, which housed 528 excerpts of speeches and images. After successive readings of the data coded in free codes, it was possible to establish a new coding process, in a hierarchical way and with links between the information, using the tree codes tool. 

Thus, the excerpts from the speeches and also the images were organized with the desired depth into three tree codes, which represent the three empirical categories of this study: 1- The perception of the professionals about adolescence, its relationships, and violence between intimate adolescent partners; 2- The reality and limits of the health practices related to adolescent VPI and 3- The place of the Third Sector in confronting adolescent VPI. The tree codes also housed 10 tree sub-codes, i.e., the sub-topics that emerged from the data analyzed in the study.

In the “professionals’ perception of adolescence, its relationships and violence between intimate partners” category, it was identified that the images most used to characterize adolescence brought the consumption of alcoholic beverages and/or illicit drugs, and appeared 23 times in the panels in all the investigated scenarios. The second most representative group of images (10 images) was the one that showed adolescence marked by the use of technology, represented by images of smartphones and notebooks. The third group of images was that which represented adolescence as a period marked by loneliness and individuality (nine images).

A unique representation of adolescence has distinguished itself in the composition of the panels. The participant’s speech reinforces the adolescent’s singularity and places them as a social subject in a certain historical period, which implies in their health-disease process and consequently in the professional performance aimed at adolescents. *I brought adolescence as a figure that brings many dishes, but each one of them has a characteristic, a specificity, although we are talking about a relatively universal phenomenon that is adolescence and the adolescent* (...) *each one of us has a different history, carries a different adolescence and so does our adolescents, each one of them is a different dish and if we label them with “everyone is the same” maybe we are wrong about that* (LMA, health sector, Curitiba).

The representation of the adolescent through images and speech has brought specific elements from the territories to which these subjects belong. The image of a smartphone with symbols representing the applications synthesized, in a participant’s perception, the vulnerabilities that mark the history of the adolescents, especially the socially excluded ones: (...) *each application on this phone has a symbol, a drug symbol, which is weed, one is a money bill, a gun, which is related to crime, a skull which is related to death* (...) (LSA, third sector, São Paulo).

Consistent with common sense, the speeches pointed to the existence of stigmata that characterize the adolescent as a carefree, lazy, and impulsive subject. Another stigmatizing element pointed to the phase of life in which irresponsible sex life begins, referred to by the groups as one of the defining characteristics of adolescence. Although the professionals realize that it is usually during adolescence that the first sexual experiences take place, it is the girls who are most criticized for behaviors associated with adolescence, for example, vanity and the first sexual intercourse. The following speeches evidence this: *Nowadays, you look at a 12-year-old girl and don’t dare to say she’s 12, she’s a girl who wants to be a grown woman, to walk down the street and draw attention* (PAF, health sector, São Paulo)*;* (...) *That’s why they* [female adolescents] *start too early their sexual life* (LZG, health sector, São Paulo). 

The professionals considered that affective and/or sexual relationships can mean to adolescents a way to socially ascend in their territory. This social escalation can occur, especially for girls, when the partner has power in the drug trafficking reality. Often, even pregnancy is desired by teenagers for a matter of status: (...) *it is a matter of status for them. Dating one of them* [drug trafficker] *getting pregnant by a trafficker* (...) *they want it!* (JEB, health sector, Curitiba).

Adolescent VPI has been described as common in the investigated scenarios, and even the use of appropriate terminology stands out to describe physical violence with terms such as: “a slap in the face”, “push”, “pinch”, and “beating”.

For the participants, adolescent VPI is the result of a power dispute process, in which there is a clear division of roles, with one assuming the role of owner and the other the role of possessed, in a subordination relationship. Teenage women were the most remembered as victims when, for example, the power of men over their behavior and outfits stands out: (...) *when you assume an intimate life with your partner. He feels like he owns you, and you have to do what he wants, dress up, look at who they want, otherwise, he beats you* (NMBS, health sector, São Paulo).

Cyberviolence has been described as a common problem in the territories, with the establishment of a conditional relationship that subjugates mainly the adolescent girl and naturalizes its occurrence, from the understanding that the materialization of cyberviolence happens due to condemnable behavior, as expressed below: (...) *we had here a time when the lads used to have* [sexual] *relations with the girls and share it on WhatsApp* (...) *the girls took pictures naked and sent them to the boys, who don’t do this like the girls* (CMS, Third Sector, São Paulo). 

The results point to the tendency of the participants to assign responsibility to someone for violent situations, namely: the adolescent herself, especially when she keeps a sexual relationship with more than one partner; the social networks and the media for conveying messages or interfering in relationships; the family, or more specifically, the mother, especially when she avoids nuclear family patterns or is headed by women who have several working hours and, according to the participants, have no time left to pay attention to their children. The following excerpt exemplifies this vision: [violence occurs] *because the mother stays out all day.* [She] *no longer has that patience to sit and talk, because then she comes home and has to do this, that and that* (CMJF, health sector, Curitiba). 

The “Reality and limits of the health practices related to adolescent VPI” empirical category showed that, in the health services of both cities, adolescent VPI is usually detected by the professionals during prenatal consultations, when they perceive physical signs of aggression or from the reports of the adolescents. In general, these adolescents are portrayed in a context of vulnerabilities, especially in relation to early pregnancy, drug addiction, and urban violence: *pregnant* [adolescent], *in prenatal consultations, had bruises and abrasions unveiled. When questioned about the marks, the excuses were stairs, slip, and domestic accidents. At the fifth appointment, she came up with clear strangulation marks. She couldn’t hide it and said, “my boyfriend tried to kill me; since I got pregnant, he spends the weekend drinking and when he gets home he takes it out on me.”* (NGM, health sector, São Paulo). 

When the professionals talk about the health practices directed at adolescents in situations of violence, they state that the assistance is guided by a biomedical perspective, directed at physical interventions, especially contraceptive measures, as exemplified in the following speech: (...) *I follow a case, a girl and a lad (...) she had two of his children, she was beaten during pregnancy and [the baby] was born prematurely* (...)*. Now, we deliver her contraception at home. Every month I go to her house to know how everything is going, but violence still exists, the lad is too violent* (JAOK, health sector, Curitiba). 

We perceive that these practices are being disaggregated from gender matters, that is, they do not recognize adolescent VPI as a gender issue, socially and historically determined. 

The following statement reveals that, in the perception of the professional, the confrontation of adolescent VPI is not the health sector’s responsibility, which is only responsible for the consequent physical harm, thus corroborating practices that can exclude the adolescent from the integrality of care: *let’s be honest, this* [VPI] *is not our responsibility. We end up taking this situation on us because, otherwise, we will receive a girl with wounds or a pregnant woman* (…) *this has nothing to do with care. We just deal with the consequence, put out the fire. The violence issue itself, that’s on the police, on the Guardianship Council, on the police station* (SNSJ, health sector, Curitiba).

In the municipality of Curitiba, the presence of the Protection Network for Children and Adolescents in a Risk Situation for Violence seems to modify the concentration pole of attention actions for adolescents in a situation of VPI. While in the São Paulo service, the actions were restricted to a few professionals and to a space in the Health Unit, in the city of Curitiba there is a more structured articulation between professional categories and different bodies that compose the Protection Network.

Part of the professionals spoke of aspects that can be understood as limitations related to the confrontation of adolescent VPI. Considering that the work process in PHC suffers a process of precariousness throughout all the national territory, we see that the testimonies sometimes translate these issues, felt and tensioned in professional daily life. The following comment explains the excessive work demand of the health professionals within PHC: (...) *we’re crammed with a lot of other services and you end up leaving it* [adolescent VPI] *aside because you don’t have time to do* (SCMM, health sector, Curitiba). 

The professionals described isolated actions adopted by the services that pointed to the possibilities of tackling adolescent VPI. It was identified that the dismantling of these actions is more a reflection of the precariousness of the services, translated into a lack of professionals and financial investment, which results in a feeling that denotes defeatism and impotence in the participants: (...) *before, we used to organize something here in the district. We had an internal contest, they [the adolescents] had to produce a media work from the year’s topic, which had been decided by the healthy teenager program coordination that existed at the time, within the Network. We had it, you know? Had. We no longer have it, we have had the service deconstructed* (RSS, health sector, Curitiba). 

The statements that composed the “The place of the Third Sector in confronting adolescent VPI” empirical category revealed that the professionals from the Third Sector in the cities of Curitiba and São Paulo indicated the presence of a pulverized adolescent VPI in the investigated territories. The most cited was the VPI perpetrated against female adolescents, being expressed mainly through sexual violence.

Some speeches were significant, of those who consider that, when acting in the confrontation of adolescent VPI, the Third Sector takes a role that should be taken by the State, translated in the mention to the city hall: (...) *we do a work that should be the city hall’s.* (LPPN, Third Sector, Curitiba)

The Third Sector professionals demonstrated unawareness of the resources and public health policies available to confront adolescent VPI. The reports show that these services often act far from the state’s practices: (...) *if I were in a situation like this, I wouldn’t know the nuclei I can go for help, or anything like that* (...) *I don’t know services* (LTI, Third Sector, São Paulo).

Significant mentions were made of the poor articulation of the Third Sector with the health sector, highlighting a feeling of not belonging to a network structure aimed at confronting violence: (...) *if we invite them, they [health sector] should participate. They do not. Why? Because they don’t have a day, because they have a meeting, because they don’t have staff. They must stop with the excuses! I made 38 invitations, I invited the BHU, the nursing, the reception, the direction, and the CHAs, nobody came* (...) *So, how do we create a Network? Like this?* (MFMS, Third Sector, São Paulo)

Another speech revealed issues about the limited visibility of the health sector interventions in the territories, reducing them to the biological and curative spheres: what *is happening today in health care? It’s not taking on its role! Health care is coming back to be like this “if you broke your arm, should go to health care, if you got cut, should go to health care, if you are vomiting, go to health care”, but violence is not its problem!* (...) *So what’s going on? The entities they call NGOs are taking over the role of the government* (MFMS, Third Sector, São Paulo). 

## Discussion

The participants portrayed adolescence based on stereotypes. This result is in line with that identified by another study which states that, among professionals who develop actions aimed at the adolescent population, the vision that associates adolescence with circumstances of crisis and rebellion prevails. In this construction, the adolescent is not considered an autonomous subject, on the contrary, there are growing visions of a regulatory nature that stigmatize and subjugate these subjects^(^ 10 ^)^, as verified in the study in question.

The territory was perceived for containing important wear potentials for adolescents, expressed by vulnerabilities. Whereas social relations are power relations, it is possible to assume that, because they hold less power than adults, adolescents become more vulnerable to processes of social exclusion that degrade their health^(^ 18 ^)^, among them, VPI.

Most of the speeches have referred to gender relations from the classical hegemonic approach, which seeks to explain such relations based on femininity and masculinity as biological, innate and therefore naturalized characteristics^(^ 12 ^)^.

The professionals attributed to the girls more severe moral judgments than to the boys regarding the behaviors evidenced in affective or sexual relationships. While adolescents of both genders are subjugated in the generational construct, girls suffer a double sanction in the perspectives of the generation and gender categories, which naturalizes the subjugated position attributed to adolescent girls and belittles their decisions and aspirations regarding affective and/or sexual relationships.

By analyzing the results in the light of the generation analysis category^(^ 13 ^)^, it is possible to understand that it determines the social space occupied by the adolescents and the construction of a polarized relationship between them and the participants in the study. On one pole there are adolescents who have their social place determined also by the experience of relationships and their gender roles; and on another pole, there are health professionals or adults from the Third Sector, who experience a generational gap responsible for subordinating adolescents and making dialog between adolescents and adults impossible^(^ 6 ^)^. 

In this study, physical violence was the most cited, which contradicts findings of another research, in which professionals from different departments who work with adolescents perceive psychological violence as the main expression of VPI in adolescence^(^ 19 ^)^. This data may be related to the invisibility and naturalization of VPI in the territory, implying greater perception of those situations that are socially typified as more serious expressions of violence. 

Another important result of this study was that the participants identified the presence and impact of violence that occurs in the digital space, the so-called cyberviolence. This manifestation of violence can be considered as or more severe than that experienced personally. More spaces and formats for the perpetration of violence can be created virtually, as it is difficult to distance oneself from the permanent recording of an offensive message and, in addition, fear prevails that the content of virtual violence, for example, an intimate photo, will become public^(^ 20 ^-^ 21 ^)^.

The social devaluation of affective and/or sexual relationships among adolescents was highlighted by the participants from both professional groups and used as an element to blame the girls for VPI. The fact that they are not inserted in socially accepted relationships, such as marriage, may erroneously translate that they can easily get rid of the situation, which does not always happen. This perception denotes the social weight of the marriage institution, understood as a scenario of acceptance and submission of women^(^ 22 ^)^.

The participants also attributed accountability on the adolescents’ mothers for the VPI situation suffered. It must be taken into account that, by attributing responsibility for violence to the mother, the gender yoke on these women is revealed, from whom one expects dedication to raising children, even if she is the head of the family and has other obligations and responsibilities related to their subsistence^(^ 23 ^)^. 

The statements of the health professionals showed that the situations of adolescent VPI identified in these services are those that have been aggravated due to social processes and, therefore, are detected in routine services, such as prenatal consultations. This condition may be related to the incipient way in which the adolescent is inserted into the health services, especially in PHC^(^ 24 ^)^. 

The results regarding the health practices revealed that they are based on a fragmented and medicalizing perspective, reproduced by the biomedical model. The State and society can act as driving forces in the process of medicalization. The existence of a network of power that crosses the entire social structure, allied to the economic order of the capitalist market, determines the weakening of society to resist medicalization. This process is as much driven by the potential for technology and innovation in health, as it is by the consumer society, also induced by the health services themselves to consume more and more pharmaceutical products^(^ 25 ^)^.

PHC professionals feel unprepared to act in adolescent VPI situations. However, we found differences between the cities of Curitiba and São Paulo, perhaps due to the historical presence of the Curitiba’s Network for the Protection of Children and Adolescent at Risk Situations of Violence. The Network’s actions impact and direct the practice, for example, by broadening the scope of actions beyond those restricted to a certain professional category.

Other limitations identified by the PHC professionals can be understood from the perspective of work precariousness. Issues like the excessive work demand and the lack of professionals - impacts verified nationally in the public health care services - and the dismantling of projects and actions considered successful in terms of adolescent health care limit professional action and cause feelings such as frustration and impotence. 

Concerning the professionals from the Third Sector, the fact that there is a gap of links with the health services prevailed. The labels that stand out in both researched scenarios are those of a health service distant from confronting the problem. A similar reality was exposed in a study which identified that, among 11 NGOs that maintained projects with the adolescent population, there was no monitoring process by inspection bodies or inter-sectoral proposals between these institutions and other public services^(^ 9 ^)^.

The professionals of the Third Sector understand that, sometimes, they assume demands that they consider should be met by the State. In a context marked by social inequality imposed by the capitalist accumulation model, organizations linked to the Third Sector are often the only option for assistance in impoverished communities, taking on activities to guarantee rights, which should be carried out by the State^(^ 26 ^)^.

The issues identified by this study revealed limitations to the formalization of inter-sectoral proposals between Third Sector institutions and the health field. For example, the poor visibility that the health service interventions have assumed in the territory and the lack of recognition of available resources for addressing adolescent VPI. This may reflect the relative invisibility of the Third Sector regarding the other State structures^(^ 9 ^,^ 26 ^)^. Furthermore, this result points to the need to advance actions for political empowerment and thus influence the public policies of Third Sector entities, recognizing their role in the maintenance of fundamental human rights in Brazil^(^ 27 ^)^. 

The limitation of this study was the difference in the number of professionals who participated in the survey in the cities of Curitiba and São Paulo and the fact that the surveyed health services were basic Health Units and with FHS; however, as it is a qualitative survey, this question is minimized in view of the wealth of data generated.

## Conclusion

The perceptions about adolescence, the experience of affective and sexual relationships, and adolescent VPI are sustained by historical and social determinations that subjugate adolescents and the relationships they maintain, depriving them of rights. This condition, restricted to superficiality, also determines the connotation of the practices in health care and in the services of the third sector investigated, spaces wherein adolescents transit daily. 

Specifically, in the health sector, we found that adolescent VPI is identified at the time of meeting other demands perceived under the biomedical hegemonic paradigm. Still in this sector, there is an important barrier to the confrontation of adolescent VPI, characterized by the precariousness of the work process in health.

When confronting the speeches produced by the participants from the health sector and the Third Sector in both municipalities, it became evident that none of the researched services seemed to take responsibility for confronting VPI. There seems to be an alternation of perceptions about the responsibility of facing adolescent VPI, without, however, any of the services actually assuming it. In this way, adolescents are impaired, as they do not have adequate programs or services to deal with the problem.

As a challenge, the need is evidenced to promote actions that consider the understanding of adolescents as socially and historically determined subjects, from their insertion in society. Indeed, to reveal the specificities of a social phenomenon such as adolescent VPI implies to visualize and advance scientific knowledge on the subject in the health field in order to strengthen alternatives that can modify health and inter-sectoral work practices and processes directed to the adolescent population, a necessary condition for the protection of this group in a context of gender equality and generational respect. 
